# Development of an RP-HPLC Method for Quantifying Diclofenac Diethylamine, Methyl Salicylate, and Capsaicin in Pharmaceutical Formulation and Skin Samples

**DOI:** 10.3390/molecules29122732

**Published:** 2024-06-08

**Authors:** Lubna H. M. AlNahwa, Hazim M. Ali, Tamer H. A. Hasanin, Khaled Shalaby, Mutairah S. Alshammari, Alaa M. Alsirhani, Sabrein H. Mohamed

**Affiliations:** 1 Department of Chemistry, College of Science, Jouf University, P.O. Box 2014, Sakaka 72388, Aljouf, Saudi Arabia; lubna.h.m.alnahwa@gmail.com (L.H.M.A.); hmali@ju.edu.sa (H.M.A.); msshamari@ju.edu.sa (M.S.A.); amassaf@ju.edu.sa (A.M.A.); 2 Department of Chemistry, Faculty of Science, Minia University, El-Minia 61519, Egypt; 3 Department of Pharmaceutics, College of Pharmacy, Jouf University, P.O. Box 2014, Sakaka 72388, Aljouf, Saudi Arabia; khshalabi@ju.edu.sa; 4 Department of Chemistry, Faculty of Science, Cairo University, Giza 12613, Egypt

**Keywords:** diclofenac diethylamine, methyl salicylate, capsaicin, Omni Hot Gel^®^, RP-HPLC

## Abstract

An RP-HPLC method with a UV detector was developed for the simultaneous quantification of diclofenac diethylamine, methyl salicylate, and capsaicin in a pharmaceutical formulation and rabbit skin samples. The separation was achieved using a Thermo Scientific ACCLAIM^TM^ 120 C_18_ column (Waltham, MA, USA, 4.6 mm × 150 mm, 5 µm). The optimized elution phase consisted of deionized water adjusted to pH = 3 using phosphoric acid mixed with acetonitrile in a 35:65% (*v*/*v*) ratio with isocratic elution. The flow rate was set at 0.7 mL/min, and the detection was performed at 205 nm and 25 °C. The method exhibits good linearity for capsaicin (0.05–70.0 µg/mL), methyl salicylate (0.05–100.0 µg/mL), and diclofenac diethylamine (0.05–100.0 µg/mL), with low LOD values (0.0249, 0.0271, and 0.0038 for capsaicin, methyl salicylate, and diclofenac diethylamine, respectively). The RSD% values were below 3.0%, indicating good precision. The overall greenness score of the method was 0.61, reflecting its environmentally friendly nature. The developed RP-HPLC method was successfully applied to analyze Omni Hot Gel^®^ pharmaceutical formulation and rabbit skin permeation samples.

## 1. Introduction

Rheumatoid arthritis affects approximately 5 out of every 1000 individuals and can result in serious joint damage and disability. There has been considerable advancement in the past 20 years in understanding the pathophysiology of the disease, identifying the best outcome measures, and developing successful treatment approaches, emphasizing the importance of early diagnosis and intervention for rheumatoid arthritis [1]. Medicines that are intended for external application are known as ointments and gels. The active pharmaceutical ingredient used in the ointment or gel is placed in an optimal chemical environment, depending on its intended use. Depending on the base, active ingredients can be dissolved, suspended, or emulsified. In cases like these, quantitative analysis is not a straightforward process. Quantification of active pharmaceutical ingredients in topical gels and ointments is typically performed through laborious extraction followed by chromatographic analysis. Near infrared, infrared, and nuclear magnetic resonance techniques are also available for this purpose [2].

**Figure 1:**
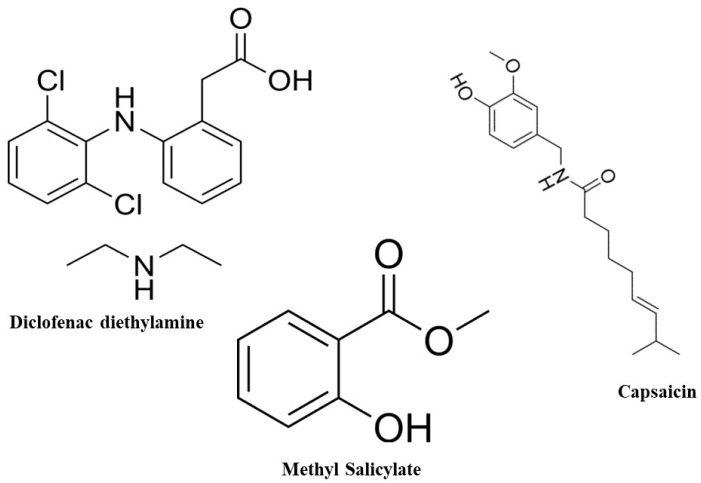
The structural formula of diclofenac diethylamine, capsaicin, and methyl salicylate.

Diethyl ammonium 2-[(2,6-dichloroanilino)phenyl]acetate, known as diclofenac diethylamine, is a non-steroidal anti-inflammatory drug (NSAID) used to relieve pain and reduce inflammation (Figure 1). The British Pharmacopoeia recommends using HPLC and HPTLC methods to analyze diclofenac [3]. Following a thorough survey, diclofenac was analyzed using the HPLC technique, either alone or in combination with other medications [4,5,6,7,8,9,10,11,12,13]. There is no previously published HPLC technique to analyze diclofenac diethylamine in a combination containing methyl salicylate and capsaicin. Diclofenac diethylamine was previously analyzed in a mixture with mephenesin [14], and curcumin [15] using HPLC. Topical sprays are gaining popularity due to their non-tacky and comfortable application compared to traditional ointments and creams. Gels, being 99% colloid immobilized by surface tension [16], are commonly used in topical formulations. Capsaicinoids, primarily capsaicin, are synthesized and stored in the placenta of Capsicum fruits. They are utilized in neurological studies to stimulate sensory neurons and alleviate bladder inflammation [17]. Figure 1 shows the structural formula of capsaicin.

Methyl salicylate, Figure 1, is an anti-inflammatory drug that may be found in a variety of over-the-counter products, including lotions and ointments for muscle aches. Methyl salicylate was used as a test substance to evaluate the effectiveness of chemical suits through a man-in-simulator testing procedure [18].

Diclofenac diethylamine, methyl salicylate, capsaicin, and menthol are the active ingredients in Omni Hot Gel^®^, which is intended to treat osteoarthritis of the joints and relieve acute musculoskeletal pain. The goal of the current study is to develop a quick and easy RP-HPLC technique to separate and quantify diclofenac diethylamine, methyl salicylate, and capsaicin in Omni Hot Gel^®^ and skin using UV detection.

## 2. Results and Discussion

### 2.1. RP-HPLC Method Optimization

The analyte retention time is influenced by the amount and concentration of the solvent employed in the mobile phase. Therefore, selecting the mobile phase in RP-HPLC analysis is integral to method optimization. The appropriate solvents were selected based on their compatibility with the sample and column stationary phase [19]. This research used a steady flow rate of 0.7 mL/min and a 205 nm detecting wavelength. To select the best mobile phase, various trials were carried out on mobile phases A and B, and deionized water acidified with phosphoric acid (pH = 3) and acetonitrile (ACN) were mixed at a ratio of 35:65% (*v*/*v*) as the optimum mobile phase. The injected concentrations of standards were 5.0 μg/mL for each drug (CS, MS, and DDEA) in the presence of MT and using IP as internal standards. The best resolution was observed in the case of utilizing deionized water acidified with phosphoric acid as mobile phase A and 100% acetonitrile as mobile phase B. Based on column resolution, maximum peak area, and short retention time, it was found that a mobile phase A is deionized water-acidified by phosphoric acid (pH = 3), and a mobile phase B is 100% acetonitrile with a ratio of 35:65% (*v*/*v*) and isocratic elution mode were the optimum mobile phase and elution mode.

**Figure 2:**
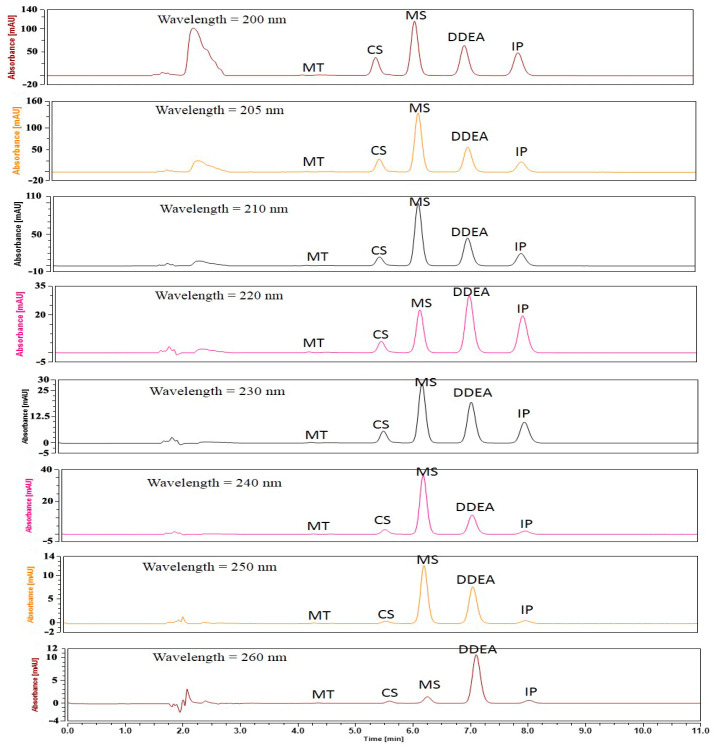
HPLC chromatograms of separation of 5 µg/mL of each capsaicin (CS), methyl salicylate (MS), and diclofenac diethylamine (DDEA) in the presence of 5 µg/mL of each MT and IP (internal standard) using phosphoric acid in water (pH = 3, mobile phase A) and 100% ACN (mobile phase B) at different wavelengths. The flow rate was adjusted at 0.7 mL/min.

**Figure 3:**
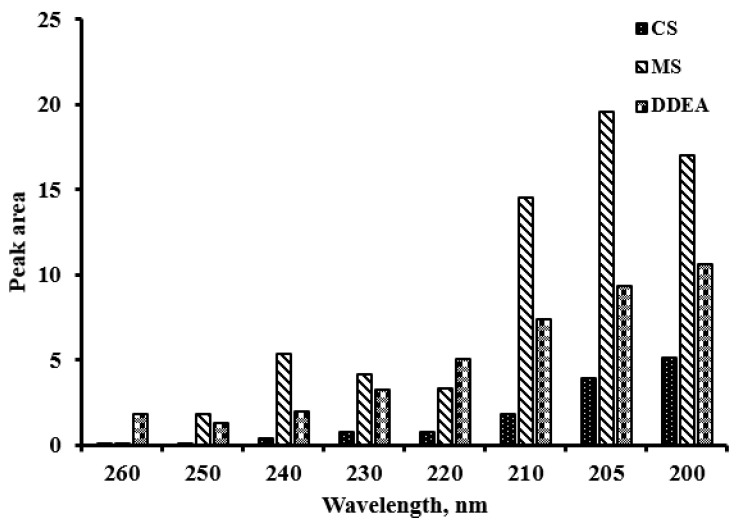
Comparison between peak areas of capsaicin (CS), methyl salicylate (MS), and diclofenac diethylamine (DDEA) at different wavelengths.

As the pH affects the sensitivity, its effect was studied by changing the pH of the mobile phase in the range of 2.5 to 5.0. It was found that the pH of the mobile phase had no substantial effect on the results. Therefore, a pH of 3 was chosen for the column’s safety. Six different flow rates were studied based on the peak area of the analyzed drugs, ranging from 0.4 to 1.2 mL/min. The optimal flow rate with good resolution and maximum peak area was determined to be 0.7 mL/min. Additionally, eight different absorption detection wavelengths (200, 205, 210, 220, 230, 240, 250, and 260 nm) were tested, with the best resolution and clear peak separation observed at 205 nm (Figure 2 and Figure 3).

**Figure 4:**
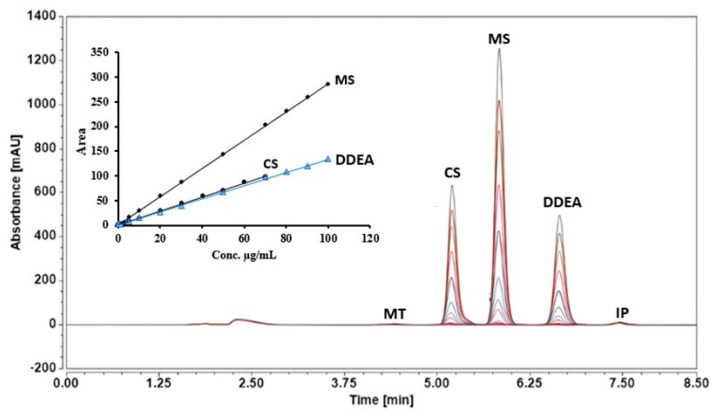
RP-HPLC chromatograms and equations of the tested compounds, capsaicin (CS), methyl salicylate (MS), and diclofenac diethylamine (DDEA), using different concentration ranges (0.05 to 100 µg/mL and 0.05 to 70 µg/mL) in the presence of 5 µg/mL of menthol (MT) and ibuprofen (IP) as internal standards at an isocratic flow of 0.7 mL/min, with phosphoric acid in water and ACN mobile phases, at 25 °C and a wavelength of 205 nm.

Furthermore, five-column temperature (20, 25, 30, 35, and 40 °C) settings were tested for optimal resolution and peak separation, with the best results observed at 25 °C. Table 1 and Figure 4 display the best conditions for the simultaneous analysis of the three cited drugs in the presence of ibuprofen and menthol as internal standards.

### 2.2. Method Validation

The proposed method was evaluated for linearity, specificity, accuracy, concentration range, precision, robustness, detection limit, repeatability, quantitation limit, and system suitability using ICH guidelines (International Conference on Harmonization) [20].

Linearity was evaluated by plotting the peak area as a function of the concentrations of standard capsaicin (CS), methyl salicylate (MS), and diclofenac diethylamine (DDEA). The calibration curve is found to be linear from 0.05 to 70 µg/mL for CS, and from 0.05 to 100 µg/mL for MS and DDEA. The linearity was confirmed by the correlation coefficient of the curve (R^2^ = 0.9994, 0.9996, and 0.9993, respectively), as shown in Table 2 and Figure 4. Upon comparing the obtained data with an acceptable correlation coefficient in ICH guidelines (0.990) [21], these correlation coefficient values revealed adequate linearity of the calibration graphs.

#### 2.2.1. Detection Limit (LOD) and Quantitation Limit (LOQ)

The detection limit (LOD) of a certain analytical process is the lowest concentration of an analyte that can be detected. The quantitation limit (LOQ), in contrast, is the lowest quantity of an analyte that can be quantitatively measured with acceptable precision and accuracy. They are critical factors in the validation process, especially when the analyte is detected in trace or ultra-trace amounts [22].

As shown in Table 2, the acquired values for CS, MS, and DDEA for LOD were determined to be 0.0294, 0.0271, and 0.0038 µg/mL, respectively, whereas the obtained values for LOQ were 0.0979, 0.0902, and 0.0127 µg/mL, respectively. The obtained data were evidence of the acceptable sensitivity of the method.

#### 2.2.2. Accuracy

It was measured by calculating the recovery to determine how close the experimental values were to the real value. Accuracy was determined using five replicates for six concentrations of the specified analyte. The tested concentrations were 0.05–70.00 µg/mL for capsaicin and 0.05–100.0 µg/mL for diclofenac diethylamine and methyl salicylate in their mixture. The average percent recoveries for CS, MS, and DDEA solutions were 93.19–105.65%, 97.80–103.80%, and 97.13–103.9 4%, respectively, and RSD% ranged from 0.145 to 2.032%. The obtained values indicate the high accuracy of the suggested method.

#### 2.2.3. Repeatability

An analytical procedure’s precision is measured by the repeatability of the entire analytical process and is expressed as the degree of closeness of the reported findings in terms of RSD%. To assess intra-day precision, five consecutive runs of the RP-HPLC system were conducted over three days using concentrations of 0.1, 0.5, 1.0, 3.0, 10.0, and 40.0 µg/mL of standard solutions of CS, MS, and DDEA. RSD% was calculated for each concentration. The obtained data ranged from 0.338% to 1.475% for CS, 0.145% to 0.821% for MS, and 0.299% to 2.032% for DDEA. The RSD% for the inter-day checks was 0.277% to 2.296% for capsaicin, 0.233% to 2.673% for methyl salicylate, and 0.248% to 2.987% for diclofenac diethylamine. Consequently, the RSD% for the current method demonstrates acceptable precision, being less than 3.0%.

### 2.3. System Suitability (SST) Testing

To evaluate the performance of the current RP-HPLC system for the simultaneous quantification of CS, MS, and DDEA, several SST parameters were measured, including resolution (R), selectivity factor (α), tailing factor (T), capacity factor (K), column efficiency (N), and HETP (theoretical plate height). The values for R, α, T, K, N, and HETP range from 5.971 to 7.743, 1.199 to 1.239, 1.04 to 1.05, 1.132 to 1.720, 15113 to 18959, and 0.0013 to 0. 0015, respectively; see Table 3. These values fall within the recommended range [21], indicating the suitability and effectiveness of the current RP-HPLC method for the identification of the three mentioned analytes.

### 2.4. Analysis of the Pharmaceutical Formulation

**Figure 5:**
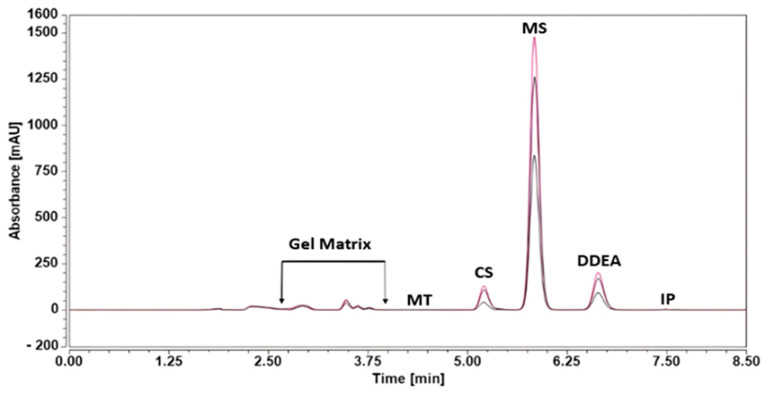
RP-HPLC chromatograms of the tested compounds, capsaicin (CS), methyl salicylate (MS), and diclofenac diethylamine (DDEA) at different doses (10.0, 50.0, and 200.0 mg dissolved in 20 mL) of methanol of Omni Hot Gel^®^ in the presence of 5 µg/mL menthol (MT) and ibuprofen (IP) as internal standard at an isocratic flow of 0.7 mL/min, with phosphoric acid in water and ACN mobile phases, at 25 °C and a wavelength of 205 nm.

The RP-HPLC-DAD method was successfully investigated for the simultaneous quantification of CS, MS, and DDEA in Omni Hot Gel^®^. According to Figure 5, there are no interferences from gel excipient in the resolution, separation, and quantification of CS, MS, and DDEA. The recovery values range from 94.39% to 100.32%, with a standard deviation percentage lower than 0.824%; Table 4 supports this point.

### 2.5. Determination of CS, MS, and DDEA in a Solution (Receptor) That Permeates through the Skin

**Figure 6:**
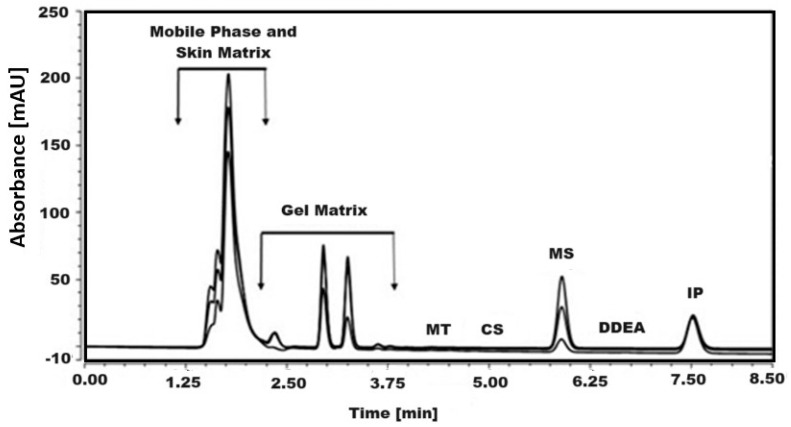
RP-HPLC chromatogram of the tested compounds, capsaicin (CS), methyl salicylate (MS), and diclofenac diethylamine (DDEA) at different doses of Omni Hot Gel^®^ permeate through skin in presence of 5 µg/mL menthol (MT) and ibuprofen (IP) as internal standard at an isocratic flow of 0.7 mL/min, with phosphoric acid in water and ACN mobile phases, at 25 °C and a wavelength of 205 nm.

The efficiency of the simultaneous quantification of CS, MS, and DDEA components of Omni Hot Gel^®^ that permeate through the skin and the effect of possible interferences on the separation region of the cited analytes were tested. According to the obtained results, there is no interference from the skin matrix in separation of CS, MS, and DDEA, as shown in Figure 6. This point confirms the efficiency of the developed RP-HPLC method in the analysis of CS, MS, and DDEA. The concentration of CS, MS, and DDEA in a solution permeated through the skin after applying Omni Hot Gel^®^ on the skin surface was checked at three time intervals and is displayed in Table 5.

### 2.6. Comparative Analysis with the Previously Published Methods

There is no previously published method for the simultaneous determination of CS, MS, and DDEA. Table 6 shows a comparison between the linearity range, LOD, and recovery of the developed RP-HPLC method and those of previously reported methods for each analyte. From a comparison with the previously published HPLC method with a UV detector for each studied compound, it was found that the LOD for capsaicin in the current study (0.00294 µg/mL) is lower than the previously published method [23], while the range (19.25–616.00 µg/mL) is wider in higher concentration for the previously published method.

For methyl salicylate, the LOD in the current study (0.0271 µg/mL) is lower than the previously published method [24], while the range for the previously published method (25.0–175.0 µg/mL) is slightly higher than the current method.

For diclofenac diethylamine, the LOD (0.0038 µg/mL) is lower than the compared published method [6,13,14], and the linear range is wider (0.05–100.00 µg/mL). These results show that the RP-HPLC method under investigation has adequate sensitivity for the simultaneous detection of CS, MS, and DDEA with a simple, fast, and accurate procedure.

### 2.7. Nature Estimation for the New RP-HPLC Method in an Eco-Friendly Way

**Figure 7:**
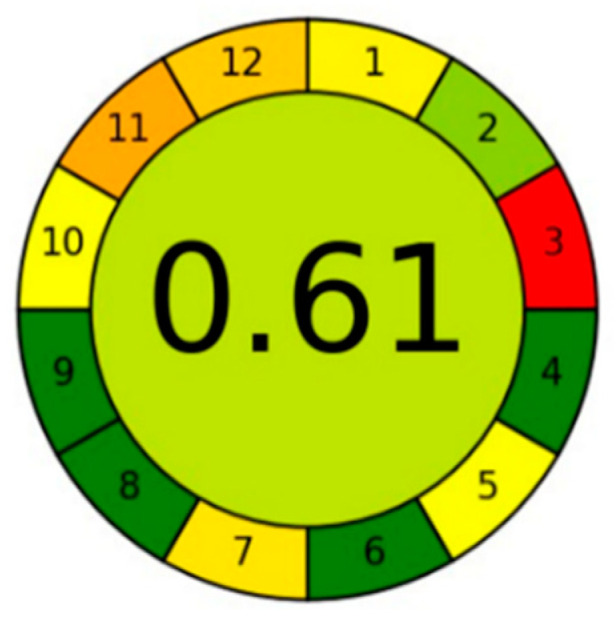
AGREE approach for estimation of method greenness for resolving a mixture of capsaicin, methyl salicylate, and diclofenac diethylamine.

The AGREE technique was chosen among green tools due to its simplicity, automation, and integration [25]. As shown in Figure 7, the new RP-HPLC method’s overall greenness score was 0.61, with a somewhat light green color in the middle of the pictogram signifying the method’s acceptable greenness quality. To accomplish the requisite separation, acetonitrile must be used, which is not ideal for technique green. Subdivisions 3 and 11 are the two primary subdivisions in the pictogram with the two lowest scores. Off-line sampling is discussed in Subdivision 3, and dangerous reagents that were utilized are discussed in Subdivision 11. The five pharmaceuticals were analyzed in 8.5 min, which only serves to further support the idea of method greenness as stated in Totally Green Subdivision 8, where several samples are determined in a single run and about 35 samples can be analyzed in an hour.

## 3. Materials and Methods

### 3.1. Chemicals and Materials

Diclofenac diethylamine (DDEA), methyl salicylate (MS), ibuprofen (IP), and menthol (MT) were provided by Sigma-Aldrich (Taufkirchen, Germany), while capsaicin (CS) was supplied by Biosynth Carbosynth (Louisville, KY, USA). Sigma-Aldrich (Germany) also provided acetonitrile (HPLC-grade), methanol (HPLC-grade), phosphoric acid, acetic acid, trifluoroacetic acid, formic acid, and phosphate buffer saline (PBS, pH 7.4). Omni Hot Gel^®^ (30 g) was purchased from CIPLA (Mumbai, India) and contains 0.025% CS, 10% MS, 1.16% DDEA, 5% MT, 3% linseed oil, and 1% (*w*/*w*) benzyl alcohol.

### 3.2. Methods and Instruments

The Thermo Scientific Dionex UltiMate 3000 HPLC^+^ system consists of a quaternary analytical pump (LPG-3400SD) with a degasser, an analytical split-loop thermostatic well plate autosampler (WPS-3000TSL), a thermostatic column compartment (TCC-3000SD), a solvent rack (SR-3000) without a degasser, and a UV–VIS detector (Waltham, MA, USA). A DELL PC-compatible computer with a ChromeleonTM 7.2 chromatography data system served as the equipment’s interface. The system utilized Thermo Scientific’s ACCLAIM^TM^ 120 C_18_ column (150 mm length, 4.6 mm inner diameter, and 5 µm particle packing) for sample separation. Permeability tests on rabbit skin samples were conducted using Franz diffusion chambers (FDCs), obtained from PermeGear (Hellertown, PA, USA).

### 3.3. Preparation of Solutions

#### 3.3.1. Standard Solutions

Standard stock solutions of diclofenac diethylamine, capsaicin, menthol, and ibuprofen were prepared by dissolving 20.0 mg of each compound in methanol. The solutions were then diluted to a concentration of 400.0 µg/mL by adding methanol to a final volume of 50.0 mL. A 1.0 mg/mL stock solution of methyl salicylate was prepared by diluting the compound with methanol to a final volume of 50.0 mL. Further dilutions were made using methanol as the solvent.

#### 3.3.2. Preparations of Omni Hot Gel^®^ Solutions

Omni Hot Gel^®^ amounts of 10.0, 50.0, 200.0, and 500.0 mg were dissolved in 20 mL of methanol (HPLC-grade) without any pretreatment to obtain varying concentrations of the target compounds.

#### 3.3.3. Preparation of Rabbit Skin

Permeability studies were conducted on rabbit skin samples using Franz diffusion cells (FDCs) purchased from PermeGear (USA). The skin samples were clamped between the donor and receptor sections, with the receptor section facing upwards. The receptor compartment contained 10 mL of PBS at pH 7.4 and was stirred continuously at 37 °C. The effective diffusion area was 3.14 cm^2^. Skin samples were mounted on the FDC and allowed to equilibrate for at least 30 min before the experiment. Omni Hot Gel^®^ was added to the donor chamber under non-occlusive conditions. The study was conducted following the Local Bioethics Committee (LCBE 6-5-42).

### 3.4. General Procedure

#### 3.4.1. Chromatographic Conditions

Throughout this work, a 10 µL injection volume and a Thermo Scientific ACCLAIM^TM^ 120 C_18_ column (4.6 × 150 mm, 5 µm) were employed. To select the best mobile phase, various trials were carried out on mobile phases A and B; see Table 7. The elution mode is an isocratic system, and the flow rate is 0.7 mL/min. The measuring wavelength is 205 nm at a temperature of 23 ± 2 °C.

#### 3.4.2. Calibration Curve

Linearity was assessed by analyzing standard solutions of capsaicin at concentrations ranging from 0.05 to 70.0 µg/mL and standard solutions of methyl salicylate and diclofenac at concentrations ranging from 0.05 to 100.0 µg/mL. Each solution was injected five times into the HPLC system to generate calibration curves by plotting the analyte peak area against concentration. The correlation coefficient (R^2^) was used to evaluate the linearity of the calibration curves.

## 4. Conclusions

A sensitive RP-HPLC-UV method was developed for the analysis of capsaicin, methyl salicylate, and diclofenac diethylamine in the presence of menthol, using ibuprofen as an internal standard. The method was validated according to ICH requirements, with a wide linear range (0.05–70.0 µg/mL for capsaicin and 0.05–100.0 µg/mL for diclofenac diethylamine and methyl salicylate) and low detection limits (0.0038, 0.0271, and 0.0294 µg/mL, respectively). The validated HPLC method has an advantage over the previously published method in that it can analyze the three compounds simultaneously for the first time with very low LOD values, is time-saving (less than 10 min per run), and is suitable for analyzing these compounds in pure solutions, pharmaceutical formulations, and rabbit skin without interference.

## Figures and Tables

**Table 1:** Optimum conditions for the separation of diclofenac diethylamine, capsaicin, and methyl salicylate in the presence of menthol and ibuprofen, simultaneously.

Column type	ACCLAIM™ 120 C_18_ column (4.6 mm × 150 mm, 5 µm sized particle packing).			
Mobile phase composition	Mobile Phase A (deionized water acidified with phosphoric acid (pH = 3)): Mobile Phase B (100% Acetonitrile), (35:65% (*V*/*V*)			
Elution mode	Isocratic mode			
Flow rate	0.7 mL/min			
Wavelength	205 nm			
Column temperature	25 °C			
Retention time (min)	Capsaicin	Methyl salicylate	Diclofenac diethylamine	Ibuprofen
	5.22	5.85	6.66	7.51

**Table 2:** Regression equations, detection limit, and quantitation limit of capsaicin, methyl salicylate, and diclofenac diethylamine.

Compound Name	Regression Equation	R^2^	Linear Range (µg/mL)	LOD (µg/mL)	LOQ (µg/mL)
Capsaicin	Y = 1.420X − 0.237	0.9994	0.05–70.00	0.0294	0.0979
methyl salicylate	Y = 2.868X + 0.218	0.9996	0.05–100.00	0.0271	0.0902
Diclofenac diethylamine	Y = 1.337X − 0.513	0.9993	0.05–100.00	0.0038	0.0127

**Table 3:** Method system suitability (SST) testing parameters.

Parameters	Result			Reference Value [21]
	Capsaicin	Methyl Salicylate	Diclofenac Diethylamine	
Resolution, R	5.971	7.74	6.696	<1.5
Tailing factor, T	1.05	1.05	1.04	<1.5–2.0 or <2.0
Selectivity factor, α	1.226	1.239	1.199	>1.0
Capacity factor, K	1.132	1.388	1.720	1.0–10.0 is acceptable
Column efficiency, N	15,113	18,959	17,080	Increment with the efficiency of the separation
HETP *	0.0015	0.0013	0.0013	The column efficiency increases with small values.

*: The equivalent height of the theoretical plate (cm per plate).

**Table 4:** Analysis of capsaicin, methyl salicylate, and diclofenac diethylamine in Omni Hot Gel^®^.

Compound	Concentration, µg/mL	Recovery * ± S.D%	RSD%
Capsaicin	0.125	94.39 ± 0.037	0.039
	0.625	96.15 ± 0.076	0.079
	2.500	99.89 ± 0.033	0.033
	6.250	96.03 ± 0.008	0.008
	30.000	99.27 ± 0.285	0.285
	50.000	99.34 ± 0.590	0.594
	70.000	98.84 ± 0.550	0.556
Methyl salicylate	1.20	97.83 ± 0.305	0.311
	5.80	98.85 ± 0.750	0.758
	29.00	100.34 ± 0.608	0.606
	50.00	100.08 ± 0.565	0.565
	80.00	98.40 ± 0.960	0.976
	100.00	98.86 ± 0.950	0.961
Diclofenac diethylamine	1.50	98.20 ± 0.824	0.839
	5.00	98.48 ± 0.412	0.418
	20.0	98.91 ± 0.353	0.357
	50.0	97.87 ± 0.156	0.160
	80.0	98.50 ± 0.880	0.893
	100.0	97.54 ± 0.950	0.974

*: Average of 5 determinations.

**Table 5:** Determination of capsaicin (CS), methyl salicylate (MS), and diclofenac diethylamine (DDEA) in solution (receptor) that permeates through the skin at different time intervals.

	Compound	Concentration * ± S.D, µg/mL		
Exit Time (hr)		Capsaicin	Methyl Salicylate	Diclofenac Diethylamine
1		0.181 ± 0.003	0.319 ± 0.012	0.385 ± 0.013
4		0.173 ± 0.002	2.32 ± 0.036	0.395 ± 0.012
6		0.173 ± 0.001	3.84 ± 0.078	0.408 ± 0.017

*: Average of 5 determinations.

**Table 6:** Comparative analysis with the previously published methods for CS, MS, and DDEA determination.

Method	Application	Linear Range, µg/mL	LOD, µg/mL	Recovery%	Ref.
Capsaicin (CS)					
HPLC-UV detection	Capsaicin-collagen sponge	19.25–616.00	2.090	90.20–98.00	[23]
RP-HPLC UV detection	Pharmaceutical formulation and skin	0.05–70.00	0.0294	94.39–99.27	C.M.
Methyl salicylate (MS)					
HPLC UV detection	Cream formulations	25.0–175.0	2.400	99.78–100.0	[24]
RPHPLC UV detection	Pharmaceutical formulation and skin	0.05–100.00	0.0271	97.83–100.08	C.M.
Diclofenac diethylamine (DDEA)					
HPLC UV detection	Pharmaceutical formulation	4.0–20.0	0.8400	99.94–100.2	[6]
HPLC-UV detection	Pharmaceutical formulation	10.0–60.0	0.2500	99.46	[14]
HPLC-UV detection	Pharmaceutical formulation	10.0–200.0	0.0125	98.13–99.7	[13]
RP-HPLC-UV detection	Pharmaceutical formulation and skin	0.05–100.00	0.0038	97.86–98.2	C.M.

C.M.: The current method.

**Table 7:** The mobile phase compositions of the RP-HPLC method.

	Mobile Phases	
Test	A	B
Test 1	Phosphoric acid in water (pH 3)	100% Acetonitrile or 100% Methanol
Test 2	Phosphoric acid in water (pH 3)	50% Methanol + 50% Acetonitrile
Test 3	0.1% Acetic acid in water	100%Acetonitrile or 100% Methanol
Test 4	0.1 Trifluoroacetic acid in water	100%Acetonitrile or 100% Methanol
Test 5	0.1 Formic acid in water	100%Acetonitrile or 100% Methanol

## Data Availability

Data are contained within the article.

## References

[1] Aletaha D., Smolen J.S. (2018). Diagnosis and Management of Rheumatoid Arthritis: A Review. JAMA.

[2] Mazurek S., Szostak R. (2016). Quantitative Analysis of Topical Gels and Ointments by FT-Raman Spectroscopy. Vib. Spectrosc..

[3] (2007). British Pharmacopoeia.

[4] Ahmed H.M., Elshamy Y.S., Talaat W., Labib H.F., Belal T.S. (2020). Simultaneous Analysis of Chlorzoxazone, Diclofenac Sodium and Tramadol Hydrochloride in Presence of Three Potential Impurities Using Validated HPLC-DAD and HPTLC Methods. Microchem. J..

[5] Emami J., Ghassami N., Talari R. (2007). A Rapid and Sensitive Modified HPLC Method for Determination of Diclofenac in Human Plasma and Its Application in Pharmacokinetic Studies. DARU J. Pharm. Sci..

[6] Goyal A., Jain S. (2007). Simultaneous Estimation of Paracetamol, Chlorzoxazone and Diclofenac Sodium in Pharmaceutical Formulation by a Novel HPLC Method. Acta Pharm. Sci..

[7] Andraws G., Trefi S. (2020). Ionisable Substances Chromatography: A New Approach for the Determination of Ketoprofen, Etoricoxib, and Diclofenac Sodium in Pharmaceuticals Using Ion—Pair HPLC. Heliyon.

[8] Nasir F., Iqbal Z., Khan A., Ahmad L., Shah Y., Khan A.Z., Khan J.A., Khan S. (2011). Simultaneous Determination of Timolol Maleate, Rosuvastatin Calcium and Diclofenac Sodium in Pharmaceuticals and Physiological Fluids Using HPLC-UV. J. Chromatogr. B.

[9] Siddiqui F.A., Arayne M.S., Sultana N., Qureshi F. (2011). Development and Validation of Stability-Indicating HPLC Method for the Simultaneous Determination of Paracetamol, Tizanidine, and Diclofenac in Pharmaceuticals and Human Serum. J. AOAC Int..

[10] Hafsa D., Chanda S., Prabhu P.J. (2011). Simultaneous HPLC Determination of Methocarbamol, Paracetamol and Diclofenac Sodium. E-J. Chem..

[11] Shaalan R.A., Belal T.S. (2013). Validated Stability-Indicating HPLC-DAD Method for the Simultaneous Determination of Diclofenac Sodium and Diflunisal in Their Combined Dosage Form. Sci. Pharm..

[12] Chandra R., Singh S., Dutt Sharma K., Alam N., Kumar S. (2013). Quantity Based Quality Estimation of Diclofenac Sodium by Reversed Phase-High Performance Liquid Chromatography Separation Method from Formulated Tablets. Pharm. Glob..

[13] Alquadeib B.T. (2019). Development and Validation of a New HPLC Analytical Method for the Determination of Diclofenac in Tablets. Saudi Pharm. J..

[14] Mulgund S.V., Phoujdar M.S., Londhe S.V., Mallade P.S., Kulkarni T.S., Deshpande A.S., Jain K.S. (2009). Stability Indicating HPLC Method for Simultaneous Determination of Mephenesin and Diclofenac Diethylamine. Indian. J. Pharm. Sci..

[15] Chaudhary H., Kohli K., Amin S., Arora S., Kumar V., Rathee S., Rathee P. (2012). Development and Validation of RP-HPLC Method for Simultaneous Estimation of Diclofenac Diethylamine and Curcumin in Transdermal Gels. J. Liq. Chromatogr. Relat. Technol..

[16] Rathod H.J., Mehta D.P. (2015). A Review on Pharmaceutical Gel. Int. J. Pharm. Sci..

[17] Chanthai S., Juangsamoot J., Ruangviriyachai C., Techawongstien S. (2012). Determination of Capsaicin and Dihydrocapsaicin in Some Chilli Varieties Using Accelerated Solvent Extraction Associated with Solid-Phase Extraction Methods and RP-HPLC-Fluorescence. E-J. Chem..

[18] Lapczynski A., Jones L., McGinty D., Bhatia S.P., Letizia C.S., Api A.M. (2007). Fragrance Material Review on Methyl Salicylate. Food Chem. Toxicol..

[19] Agrahari V., Bajpai M., Nanda S. (2013). Essential Concepts of Mobile Phase Selection for Reversed Phase HPLC. Res. J. Pharm. Technol..

[20] Jenke D.R. (1996). Chromatographic Method Validation: A Review of Current Practices and Procedures. I. General Concepts and Guidelines. J. Liq. Chromatogr. Relat. Technol..

[21] Sahoo C.K., Sudhakar M., Ramana D.V., Satyanarayana K., Panda K.C. (2018). Validation of Analytical Procedures-A Review. Asian J. Pharm. Anal..

[22] Belter M., Sajnóg A., Barałkiewicz D. (2014). Over a Century of Detection and Quantification Capabilities in Analytical Chemistry–Historical Overview and Trends. Talanta.

[23] Guo C.-L., Chen H.-Y., Cui B.-L., Chen Y.-H., Zhou Y.-F., Peng X.-S., Wang Q. (2015). Development of a HPLC Method for the Quantitative Determination of Capsaicin in Collagen Sponge. Int. J. Anal. Chem..

[24] Shabir G.A., Bradshaw T.K. (2011). Development and Validation of a Liquid Chromatography Method for the Determination of Methyl Salicylate in a Medicated Cream Formulation. Turk. J. Pharm. Sci..

[25] Gamal M., Ali H.M., Abdelfatah R.M., Magdy M.A. (2019). A Green Approach for Simultaneous Analysis of Two Natural Hepatoprotective Drugs in Pure Forms, Capsules and Human Plasma Using HPLC-UV Method. Microchem. J..

